# Fracture in Asian Women with Breast Cancer Occurs at Younger Age

**DOI:** 10.1371/journal.pone.0075109

**Published:** 2013-09-12

**Authors:** Chun-Hao Tsa, Chih-Hsin Muo, Huey-En Tzeng, Chih-Hsin Tang, Horng-Chang Hsu, Fung-Chang Sung

**Affiliations:** 1 Graduate Institute of Medicine, China Medical University, Taichung, Taiwan; 2 Department of Orthopedics, China Medical University Hospital, Taichung, Taiwan; 3 Management Office for Health Data, China Medical University Hospital, Taichung, Taiwan; 4 Department of Public health, China Medical University, Taichung, Taiwan; 5 Division of Hematology/Oncology, Taichung Veterans General Hospital, Taichung, Taiwan; 6 Department of Pharmacology, School of Medicine, China Medical University, Taichung, Taiwan; Harvard Medical School, United States of America

## Abstract

**Background:**

Western breast cancer survivors have an increased risk of osteoporosis and bone fracture. Breast cancer occurs 10 to 20 years earlier in Asian women than in Western women. We investigated if younger Asian women with breast cancer also have increased risk of fracture.

**Methods:**

We used the universal insurance claims data from 2000 to 2003 to identify 22,076 patients with breast cancer and 88,304 women without cancer, frequency matched with age and index date (the date for a health care visit). The incidence of fracture in both cohorts and the hazard ratios (HRs) of fracture in the cancer cohort were estimated by the end of 2009.

**Results:**

The incidence of all types of fracture was higher in the breast cancer cohort than in the comparison cohort (46.72 vs. 42.52 per 10,000 person-years), with adjusted HRs (aHRs) of 1.18 (95% confidence intervals [CI], 1.03–1.35) for hip fractures, 1.12 (95% CI, 0.98–1.28) for forearm fractures and 1.24 (95% CI, 1.04–1.48) for vertebral fractures. The aHRs were significant in both non-traumatic fractures (1.29; 95% CI, 1.11–1.51) and traumatic fractures (1.12; 95% CI, 1.01–1.23). The age-specific aHR was higher for younger breast cancer patients, and was significant for <50 years old patients in both traumatic (aHR 1.35; 95% CI 1.08–1.68) and non-traumatic (aHR, 1.72; 95% CI, 1.21–2.44) fractures.

**Conclusion:**

This study suggests that Asian women with breast cancer might have an increased risk of fracture.

## Introduction

Both breast cancer and osteoporosis are disorders primarily associated with aging in women, and have been a medical challenge worldwide. Osteoporosis and the associated fractures have become important global public health issues. Almost 56 million people were diagnosed with various types of fracture in 2000, with approximately 9 million new osteoporotic fractures occur annually [1]. The incidence of breast cancer has increased globally over the past few decades [2], [3], with greater increase observed in Asian populations [4]. However, no apparent biological difference in the disease has been found between Asian and Western women [4]. Previous studies have noted that breast cancer survivors are at an increased risk of osteoporosis [5] and fracture [6]. The elevated risk of fracture in patients with breast cancer has been attributed to the effects of chemotherapy, ovarian failure, early menopause, and the use of aromatase inhibitors (AI) [7], [8], [9]. However, most clinical trials or cohort studies on fractures associated with breast cancer have been performed on Caucasian postmenopausal patients [6]. The association in other ethnic groups may be significantly different. For instance, basal bone mineral density (BMD) and the incidence of bone fracture differ among ethnic groups [10]. Even without significant biological difference in breast cancer, the incidence of breast cancer in Asian women peaks in the age of 40–50 years, whereas in Western women it peaks in the age of 60 to 70 [4]. Whether Asian women with breast cancer are also at elevated risk of fracture and if fractures occur in the younger age groups should be investigated. We, therefore, used Taiwan’s National Health Insurance (TNHI) claims data to assess the relationship using a retrospective cohort study.

## Materials and Methods

### Data Source

TNHI is a universal health insurance system established in 1995 by the Department of Health of Taiwan. By the end of 2010, over 99.9% (23.07 out of 23.162 million) of the population had enrolled in this program (http://www.nhi.gov.tw). This study used the inpatients dataset and catastrophic illness dataset established by the National Health Research Institutes (NHRI) of Taiwan for the period of 2000 to 2009 to investigate the fracture risk in breast cancer survivors in Taiwan. We used the International Classification of Diseases, 9th Revision, Clinical Modification (ICD-9-CM) to identify physician-diagnosed diseases in the claims data. This study was approved by the Ethics Review Board of China Medical University (CMU-REC-101-012).

### Study Subjects

From the catastrophic illness dataset, we identified 22,812 women with newly diagnosed breast cancer (ICD-9-CM 174), who are free from other cancers and are aged 20 years and above in 2000–2003. The diagnosis date of breast cancer was used as the index date. Women with history of hip, distal forearm, and vertebral fracture at the baseline or those who have these types of fracture within one month after the index date were excluded from the study. A total of 21,952 women were included in the breast cancer cohort. Among the women without any cancer, we randomly selected 87,808 women as non-cancer comparison cohort, and frequency matched with age and index date (the date for a health care visit). Both cohorts were followed up until the end of 2009. The subsequent fractures including the hip (ICD-9-CM 820), vertebrae (ICD-9-CM 806.20-806.9, forearm (ICD-9-CM 813) and the other type of fractures (ICD-9-CM 800–806.5, 807–812,814–819, and 821–829) were investigated.

### Statistical Analyses

Data analysis first measured the annual incidence of osteoporosis-related fracture by the type of fracture in women with breast cancer. We compared the distributions of age, location of subject’s residential area and the history of non-osteoporosis fracture, between the breast and comparison cohorts. Each study subject was followed up from the index date to the event when the fracture was diagnosed, or the date censored for loss to follow-up, death, termination of insurance, or the end of 2009. The person-years of follow-up were measured for all subjects and the incidence density was estimated by per 10,000 person-years during the follow-up period. We estimated the incidence rates of fractures for both cohorts. Hazard ratio (HR) and 95% confidence interval (CI) associated with fracture were estimated using Cox proportional hazards regression analysis. The multivariable analysis model estimated adjusted HR (aHR) controlling for age at the index date (<50, 50–64, and ≥65 years), the residential area, and non-osteoporosis fracture history, etc. We also assessed the HRs of fracture among age groups to evaluate if the fracture risk was higher for a specific age group. All analyses were performed used SAS statistical package (SAS institute Inc., Cary, NC. Version 9.1), and the significance level was set at 0.05.

## Results

### Annual Osteoporosis-related Fracture Incidence in Breast Cancer Cohort

**Figure 1:**
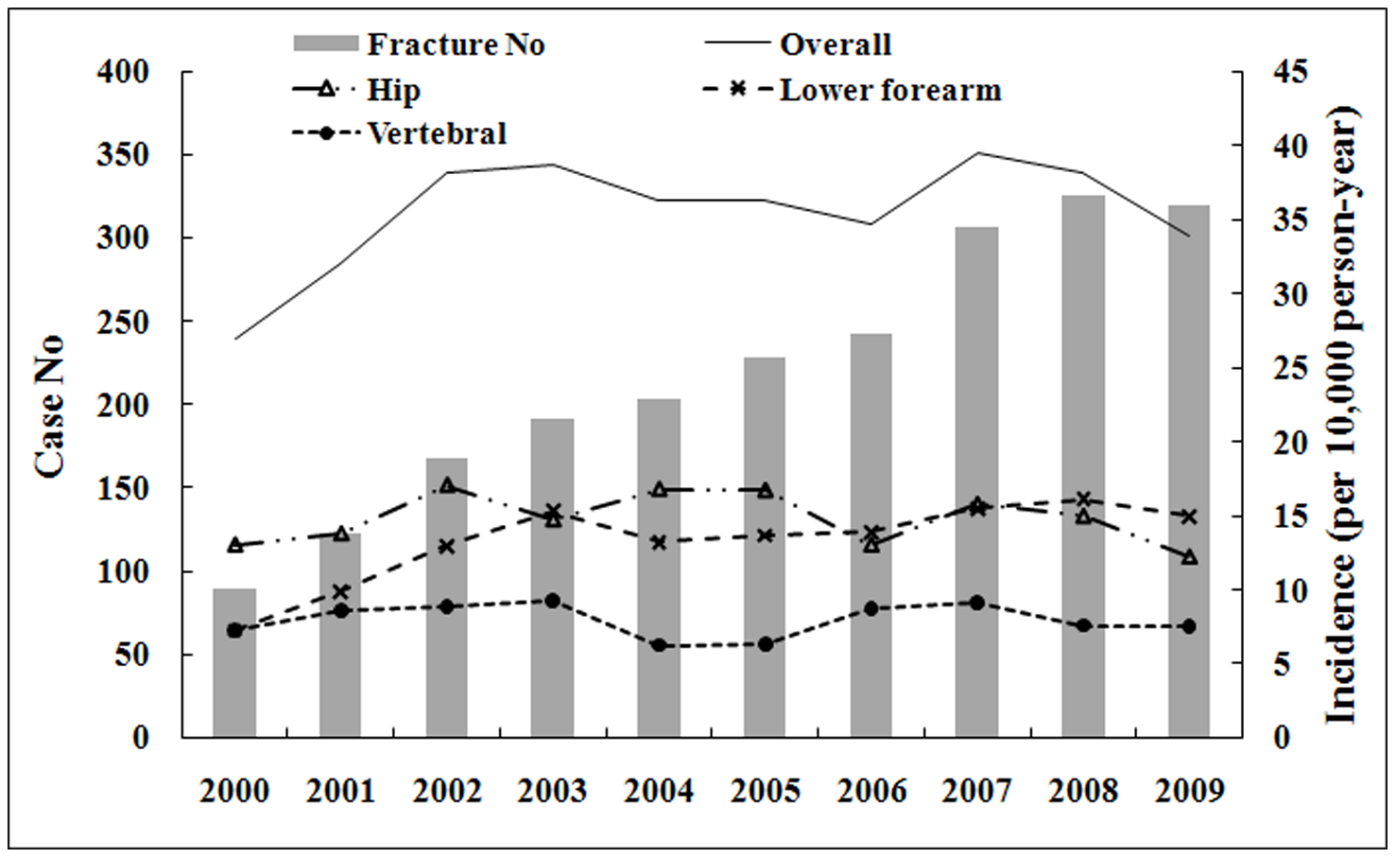
Annual incidence numbers and site specific incidence rates of osteoporosis-related fracture in women with breast cancer from 1998 to 2009.

**Figure 2:**
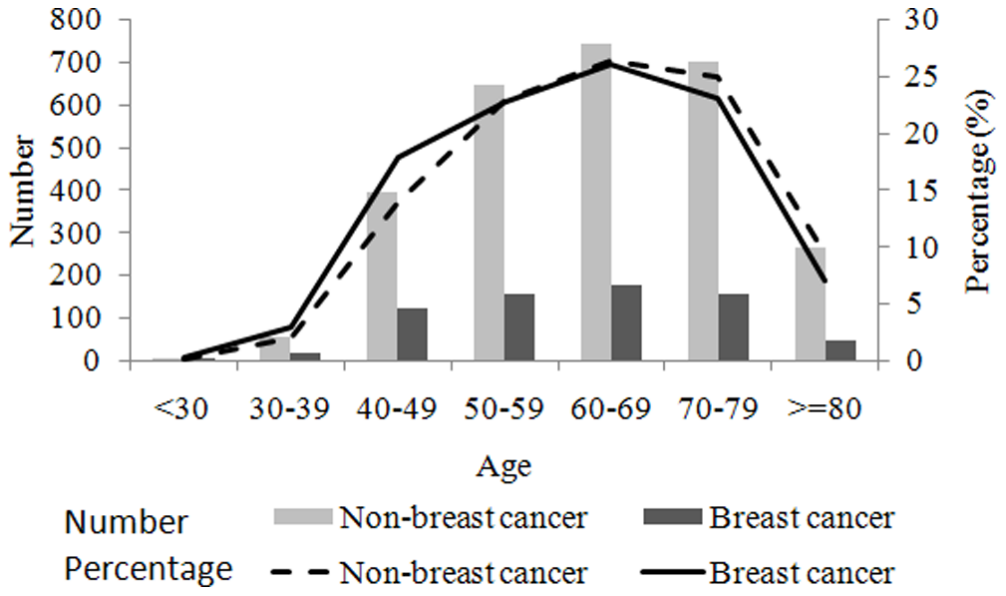
Age specific distrbution of all osteoporosis-related fracture compared between breast and comparison cohorts from 2000 to 2009.

Figure 1 shows that the annual incident cases of fracture in the breast cancer cohort increased from 89 in 2000 to 319 in 2009. The overall annual incidence decreased in 2000 and then increased to a plateau from 2002. The average overall incidence of the 3 types of fractures studied was 35.4 per 10,000 person-years, and it was higher for hip fractures than for distal forearm and vertebral fractures (14.80, 13.28, and 7.96 per 10,000 person-years, respectively). The overall age-specific incidence of fracture in the 10 years peaked during 60–69 years of age in both breast cancer and comparison cohorts (Figure 2).

### Characteristics of Study Cohorts

The baseline mean age was slightly higher in the breast cancer cohort than in the comparison cohort (51.4 (SD 12.0) vs. 51.2 (SD 12.3) years) (p = 0.02) (Table 1). Higher portion of study subjects resided in northern Taiwan, which is more urbanized than other areas. Non-osteoporosis-related fracture history (included hip, vertebral and distal forearm) was less prevalent in the breast cancer cohort than in the comparison cohort (1.62 vs. 1.94%, p = 0.002).

**Table 1:** Demographic status and fracture history compared between breast cancer cohort and comparison cohort.

		Comparison N = 87,808		Breast cancer N = 2,1952		
Variable		n	%	N	%	*p*-value
Age, year	<50	54,708	52.1	11,427	52.1	>0.99^a^
	50–64	29,148	33.2	7,287	33.2	
	≥65	12,952	14.8	3,238	14.8	
	Mean (SD)	51.2	(12.3)	51.4	(12.0)	0.02^b^
Area	Northern	39285	44.7	10,706	48.8	<0.0001^a^
	Center	17,537	20.0	4,108	18.7	
	Southern	26,547	30.2	6,195	28.2	
	Eastern and island	4,435	5.05	943	4.30	
Fracture history*		1,706	1.94	355	1.62	0.002^a^

a Chi-square test for categorical variables and ^b^t-test for continuous variables.

* Non-osteoporosis related fracture (included hip, vertebral and distal forearm) at baseline.

### Hazard Ratio and Incidence of Osteoporosis-related Fracture in the Patients with Breast Cancer

The incidence of subsequent fractures was 1.10-fold higher in the breast cancer cohort than in the comparison cohort (46.72 vs. 42.52 per 10,000 person-years), with an aHR of 1.16 (95% CI 1.07–1.27) (Table 2). The site-specific data showed significant differences for hip (aHR, 1.18; 95% CI, 1.03–1.35) and vertebral fractures (aHR, 1.24; 95% CI, 1.04–1.48) after controlling for age, area of residence, and other fracture history. The stratified analysis further showed a higher incidence of traumatic fracture than non-traumatic fracture in both cohorts (Table 2). When compared to subjects without the breast cancer, however, the breast cancer patients had significant adjusted hazard ratios for non-traumatic hip and forearm vertebral fractures, not for the traumatic fractures, particularly for hip and forearm vertebral fractures.

**Table 2:** Site specific incidence of fracture and Cox model estimated hazard ratios and 95% confidence intervals of fracture for breast cancer cohort compared to comparison cohort.

		Compared group		Breast cancer group		Crude		Adjusted	
Variable		Case	IR	Case	IR	HR	(95% CI)	HR	(95% CI)
Overall	All	2,813	42.52	681	46.72	1.11	(1.02–1.20)*	1.16	(1.07–1.27)***
	Hip	1,107	16.73	271	18.59	1.12	(0.98–1.28)	1.18	(1.03–1.35)*
	Distal forearm	1,162	17.57	273	18.73	1.08	(0.95–1.23)	1.12	(0.98–1.28)
	Vertebral	588	8.89	151	10.36	1.17	(0.98–1.40)	1.24	(1.04–1.48)*
Traumatic fracture	All	2,087	31.55	478	32.79	1.05	(0.95–1.16)	1.12	(1.01–1.23)*
	Hip	804	12.15	180	12.35	1.03	(0.87–1.21)	1.10	(0.94–1.29)
	Distal forearm	884	13.36	207	14.20	1.07	(0.92–1.25)	1.13	(0.97–1.31)
	Vertebral	431	6.52	102	7.00	1.08	(0.87–1.34)	1.15	(0.93–1.43)
Non-traumatic fracture	All	726	10.97	203	13.93	1.28	(1.09–1.49)**	1.29	(1.11–1.51)**
	Hip	303	4.58	91	6.24	1.37	(1.08–1.73)**	1.37	(1.08–1.74)**
	Distal forearm	278	4.20	66	4.53	1.09	(0.83–1.43)	1.09	(0.84–1.43)
	Vertebral	157	2.37	49	3.36	1.42	(1.03–1.96) *	1.48	(1.07–2.04)*

IR, incidence rate, per 10,000 person-years.

Adjusted model: adjusted for age, area and facture history (expect osteoporosis-related fractures (included hip, vertebral and distal forearm).

* p<0.05, **p<0.01, ***p<0.001.

### Age-specific Fracture

The age-specific incidence of fracture increased with age in both cohorts, with the peak appeared in the 60–69 ages group in both the breast cancer cohort and comparison cohort (Figure 2). The average age at which a fracture occurred in the breast cancer group was approximately 2 years younger than that in the comparison cohort (66.0±12.7 years vs. 67.9±12.6 years, p = 0.0006; data not shown).

Relative to the comparison cohort, the risk of all fractures in the breast cancer cohort was significantly higher in those aged <50 years (aHR, 1.44; 95% CI, 1.19–1.74) (Table 3). In the site-specific analysis, breast cancer patients aged <50 years were at the greatest risk of hip (aHR 1.97, 95% CI, 1.23–3.15) and vertebral (aHR, 1.44; 95% CI, 1.14–1.82) fractures. Those aged 50–64 years also had a significant risk for hip (aHR, 1.61; 95% CI, 1.24–2.10) and distal forearm (aHR, 1.43; 95% CI, 1.07–1.92) fractures. Further data analysis measured the age-specific breast cancer cohort to comparison cohort hazard ratios for traumatic and non-traumatic fractures. The risk was particularly strong for women aged <50 years for non-traumatic hip fracture with an aHR of 5.32 (95% CI, 2.30–12.3).

**Table 3:** Cox model estimated age and site specific hazard ratios and 95% confidence intervals of fracture events in breast cancer cohort compared to comparison cohort.

			Age, years								
		<50			50–64			≥65			
Variable			HR	(95% CI)		HR	(95% CI)		HR	(95% CI)	
Overall	All		1.44	(1.19–1.74)***		1.19	(1.03–1.36)*		1.04	(0.91–1.18)	
	Hip		1.97	(1.23–3.15)**		1.61	(1.24–2.10)***		0.99	(0.84–1.17)	
	Distal forearm		1.16	(0.76–1.76)		1.43	(1.07–1.92)*		1.12	(0.86–1.47)	
	Vertebral		1.44	(1.14–1.82)**		0.98	(0.80–1.19)		1.09	(0.82–1.44)	
Traumatic fracture	All		1.35	(1.08–1.68)**		1.15	(0.98–1.35)		0.99	(0.85–1.15)	
	Hip		1.25	(0.68–2.31)		1.39	(1.01–1.92)*		0.99	(0.81–1.21)	
	Distal forearm		1.45	(1.10–1.90)**		1.04	(0.83–1.29)		0.99	(0.71–1.38)	
	Vertebral		1.16	(0.71–1.90)		1.33	(0.94–1.88)		0.99	(0.71–1.39)	
Non-traumatic fracture	All		1.72	(1.21–2.44)**		1.30	(0.99–1.71)		1.16	(0.92–1.46)	
	Hip		5.32	(2.30–12.3)***		2.30	(1.43–3.67)***		1.01	(0.75–1.36)	
	Distal forearm		1.41	(0.89–2.23)		0.79	(0.51–1.22)		1.42	(0.84–2.42)	
	Vertebral		1.14	(0.50–2.60)		1.72	(1.00–2.96)*		1.46	(0.93–2.32)	

Adjusted model: adjusted for area and fracture history (expect osteoporosis-related fractures (included hip, vertebral and distal forearm).

* p<0.05, **p<0.01, ***p<0.001.

Interaction tests between age group and cancer were p<0.05 in all fracture location.

## Discussion

Our study on breast cancer in relation to fractures yielded an aHR of 1.16 for the cancer patients versus non-cancer comparison women after controlling for covariates. The incidence of traumatic fractures was greater than that of non-traumatic fractures. But, the breast cancer cohort to comparison cohort aHR estimate was significant for non-traumatic fractures not for traumatic fractures. In a case-control study, Newcomb et al. reported women with breast cancer are 20% less likely to have the history of fracture [11]. Our study also showed that women with breast cancer are less likely to have previous fracture history at the baseline compared with those without breast cancer. We also found women in Taiwan have their breast cancer and fracture occurred at younger ages than Western women. The aHRs were stronger for younger breast cancer women, particularly for the non-traumatic hip fracture with an aHR of 5.32. These data indicate a greater relative impact on non-traumatic events for women with the cancer.

Postmenopausal women with high bone density have an elevated risk of breast cancer [12]–[16], but have a lower risk of bone fracture [11]. Studies have shown that postmenopausal women with longer or higher estrogen exposure are associated with increased BMD, and increased breast cancer as well [17]–[19]. In addition, factors regulating the ossification process, such as insulin, insulin-like growth factor type 1, insulin-like growth factor type 2, and insulin-like growth factor binding protein 3, may also have association with the breast cancer risk [20]–[24].

However, breast cancer accelerates bone loss in patients. During natural menopause, women may suffer from bone loss for 3% per year in the earlier two years, and slows down to approximately 1% annually thereafter [25]. In women with breast cancer, the osteoclastic activity is increased by releasing transforming growth factors [6], even in the absence of bone metastases. Breast cancer treatment may thus enhance bone loss in women undergoing a natural menopause. Furthermore, breast cancer women with postmenopausal estrogen deficiency are at an elevated risk of bone loss with age. The use of estrogen-depleting therapies, such as third-generation aromatase inhibitors (AI), accelerates age- and menopause-related BMD loss [26]–[31]. Chemotherapy may induce ovarian failure or ovarian function suppression and cause low estrogen levels caused, leading to bone loss [5].

Chen et al. [6] reported a higher risk of fracture for breast cancer survivors in Women's Health Initiative Observational Study (WHI). However, their study population and evaluation method are different from those in our study. The breast cancer patients in our study were mainly premenopausal breast cancer women and who were younger. Moreover, most breast cancer patients were screened after fifty years old for postmenopausal women diagnosed in the WHI study [6]; by contrast, more than 50% of breast cancer patients were diagnosed under the age of 50 in premenopausal women in this study. Oriental women have an elevated risk of premenopausal breast cancer and are at the risk of bone loss, which is associated with cancer treatment [32]. For example, ovarian suppression with goserelin in premenopausal women decreases BMD by 6% to 10% during the first two years of treatment [33]. Moreover, an Austrian study found that women with the goserelin-induced ovarian suppression on AIs medication may have 17.3% enhanced BMD loss within 3 years [34]. Ovarian ablation (either medical or surgical) leads to increased bone loss in premenopausal women. Women with premenopausal breast cancer may thus have lower BMD later in their life and are at an increased risk of osteoporosis, compared with those without premenopausal breast cancer [35], [36].

Since the early premenopausal age, these women suffer not only losing trabecular connectivity in cancellous bone structural, and cortical thinning and porosity, but also experiencing reduced toughness of bone and the resistant to crack propagation. Pores coalesce and the low bone mass cannot absorb energy radiating from a fall. Hip and distal forearm fractures occurred more frequently in the present study, and hip fracture was the worst non-traumatic osteoporosis-related fractures. Therefore, the higher risk of hip fracture in women of younger age in our study groups may be related to breast cancer.

This study has several limitations. First, a few minor subclinical vertebral or wrist fractures do not systematically lead to medical management or hospitalization. The claims data also included few self-reported fractures. The incidence of vertebral fracture is low in our study and the findings are likely underestimated because of subclinical status. This inference can be verified in further analysis that compared traumatic and non-traumatic events (Table 3). Women with breast cancer have a higher risk of vertebral fracture among non-traumatic fracture probably because they visit clinics more often and probably have these subclinical events identified. However, the difference is no more significant in the analysis by age, probably because of the small sample size. Furthermore, studies have shown that self-reported fracture is generally reliable [37]–[39]. Women with breast cancer are at higher risk than the general population. Second, insurance claims files do not provide information on cancer stages. We were unable to determine if patients with advanced stages of the cancer are at considerably greater risk of fracture. Third, information on lifestyle, such as alcohol consumption and smoking, is also unavailable in the claims file; thus, we were unable to assess the association between fracture and lifestyle factors. However, lifestyle is probably not an important factor in this study because only approximately 4% of the women in Taiwan smoke [40].

The 10-year overall survival of patients with breast cancer is 75% in Taiwan [4]. As the fracture is an important factor that affects the quality of life, a multidisciplinary treatment team of breast cancer should encompass the issue of bone protection and fracture prevention, such as regular examination of BMD [41], early use of antiresorptive agents [36], [41], [42], or fall prevention facility, to improve the quality of life of young breast cancer survivors.

To the best of our knowledge, this study is the first national population-based report on fracture risk among Asian breast cancer survivors. This study suggests that women with breast cancer, particularly those diagnosed at a relatively early age, below 50 years old, should undergo prophylactic treatment to counter the increased risk of fractures.

## References

[1] Johnell O, Kanis JA (2006) An estimate of the worldwide prevalence and disability associated with osteoporotic fractures. Osteoporos Int 17: 1726–1733. 16983459 10.1007/s00198-006-0172-4

[2] Hortobagyi GN, de la Garza Salazar J, Pritchard K, Amadori D, Haidinger R, et al (2005) The global breast cancer burden: variations in epidemiology and survival. Clin Breast Cancer 6: 391–401. 16381622 10.3816/cbc.2005.n.043

[3] Anderson BO, Jakesz R (2008) Breast cancer issues in developing countries: an overview of the Breast Health Global Initiative. World J Surg 32: 2578–2585. 18283512 10.1007/s00268-007-9454-z

[4] Leong SP, Shen ZZ, Liu TJ, Agarwal G, Tajima T, et al (2010) Is breast cancer the same disease in Asian and Western countries? World J Surg 34: 2308–2324. 20607258 10.1007/s00268-010-0683-1 PMC2936680

[5] Ramaswamy B, Shapiro CL (2003) Osteopenia and osteoporosis in women with breast cancer. Semin Oncol 30: 763–775. 14663777 10.1053/j.seminoncol.2003.08.028

[6] Chen Z, Maricic M, Bassford TL, Pettinger M, Ritenbaugh C, et al (2005) Fracture risk among breast cancer survivors: results from the Women's Health Initiative Observational Study. Arch Intern Med 165: 552–558. 15767532 10.1001/archinte.165.5.552

[7] Khan MN, Khan AA (2008) Cancer treatment-related bone loss: a review and synthesis of the literature. Curr Oncol 15: S30–40. 18231646 10.3747/co.2008.174 PMC2216420

[8] Jacoby VL, Grady D, Wactawski-Wende J, Manson JE, Allison MA, et al (2011) Oophorectomy vs ovarian conservation with hysterectomy: cardiovascular disease, hip fracture, and cancer in the Women's Health Initiative Observational Study. Arch Intern Med 171: 760–768. 21518944 10.1001/archinternmed.2011.121

[9] Neuner JM, Yen TW, Sparapani RA, Laud PW, Nattinger AB (2011) Fracture risk and adjuvant hormonal therapy among a population-based cohort of older female breast cancer patients. Osteoporos Int 22: 2847–2855. 21170643 10.1007/s00198-010-1493-x PMC3166362

[10] Cauley JA (2011) Defining ethnic and racial differences in osteoporosis and fragility fractures. Clin Orthop Relat Res 469: 1891–1899. 21431462 10.1007/s11999-011-1863-5 PMC3111798

[11] Newcomb PA, Trentham-Dietz A, Egan KM, Titus-Ernstoff L, Baron JA, et al (2001) Fracture history and risk of breast and endometrial cancer. Am J Epidemiol 153: 1071–1078. 11390325 10.1093/aje/153.11.1071

[12] Ganry O, Tramier B, Fardellone P, Raverdy N, Dubreuil A (2001) High bone-mass density as a marker for breast cancer in post-menopausal women. Breast 10: 313–317. 14965600 10.1054/brst.2000.0247

[13] Cauley JA, Lucas FL, Kuller LH, Vogt MT, Browner WS, et al (1996) Bone mineral density and risk of breast cancer in older women: the study of osteoporotic fractures. Study of Osteoporotic Fractures Research Group. JAMA 276: 1404–1408. 8892715

[14] Zhang Y, Kiel DP, Kreger BE, Cupples LA, Ellison RC, et al (1997) Bone mass and the risk of breast cancer among postmenopausal women. N Engl J Med 336: 611–617. 9032046 10.1056/NEJM199702273360903

[15] van der Klift M, de Laet CE, Coebergh JW, Hofman A, Pols HA (2003) Bone mineral density and the risk of breast cancer: the Rotterdam Study. Bone 32: 211–216. 12667548 10.1016/s8756-3282(02)00972-9

[16] Kerlikowske K, Shepherd J, Creasman J, Tice JA, Ziv E, et al (2005) Are breast density and bone mineral density independent risk factors for breast cancer? J Natl Cancer Inst 97: 368–374. 15741573 10.1093/jnci/dji056

[17] Steinberg KK, Thacker SB, Smith SJ, Stroup DF, Zack MM, et al (1991) A meta-analysis of the effect of estrogen replacement therapy on the risk of breast cancer. JAMA 265: 1985–1990. 1826136

[18] Greendale GA, Edelstein S, Barrett-Connor E (1997) Endogenous sex steroids and bone mineral density in older women and men: the Rancho Bernardo Study. J Bone Miner Res 12: 1833–1843. 9383688 10.1359/jbmr.1997.12.11.1833

[19] Qu X, Zhang X, Qin A, Liu G, Zhai Z, et al.. (2013) Bone mineral density and risk of breast cancer in postmenopausal women. Breast Cancer Res Treat. 10.1007/s10549-013-2431-3 23381744

[20] Malpe R, Baylink DJ, Linkhart TA, Wergedal JE, Mohan S (1997) Insulin-like growth factor (IGF)-I, -II, IGF binding proteins (IGFBP)-3, -4, and -5 levels in the conditioned media of normal human bone cells are skeletal site-dependent. J Bone Miner Res 12: 423–430. 9076585 10.1359/jbmr.1997.12.3.423

[21] Gunter MJ, Hoover DR, Yu H, Wassertheil-Smoller S, Rohan TE, et al (2009) Insulin, insulin-like growth factor-I, and risk of breast cancer in postmenopausal women. J Natl Cancer Inst 101: 48–60. 19116382 10.1093/jnci/djn415 PMC2639294

[22] Adami S, Zivelonghi A, Braga V, Fracassi E, Gatti D, et al (2010) Insulin-like growth factor-1 is associated with bone formation markers, PTH and bone mineral density in healthy premenopausal women. Bone 46: 244–247. 19853071 10.1016/j.bone.2009.10.011

[23] Key TJ, Appleby PN, Reeves GK, Roddam AW (2010) Insulin-like growth factor 1 (IGF1), IGF binding protein 3 (IGFBP3), and breast cancer risk: pooled individual data analysis of 17 prospective studies. Lancet Oncol 11: 530–542. 20472501 10.1016/S1470-2045(10)70095-4 PMC3113287

[24] Qiu J, Yang R, Rao Y, Du Y, Kalembo FW (2012) Risk factors for breast cancer and expression of insulin-like growth factor-2 (IGF-2) in women with breast cancer in Wuhan City, China. PLoS One 7: e36497. 22662119 10.1371/journal.pone.0036497 PMC3360739

[25] Osteoporosis Update 1997. Symposium proceedings. Osaka, Japan, 13–16 November 1997: Osteoporos Int. 7 Suppl 31–222. 9565455

[26] Brufsky A (2006) Management of cancer-treatment-induced bone loss in postmenopausal women undergoing adjuvant breast cancer therapy: a Z-FAST update. Semin Oncol 33: S13–17. 16730272 10.1053/j.seminoncol.2006.03.022

[27] Rabaglio M, Sun Z, Price KN, Castiglione-Gertsch M, Hawle H, et al (2009) Bone fractures among postmenopausal patients with endocrine-responsive early breast cancer treated with 5 years of letrozole or tamoxifen in the BIG 1–98 trial. Ann Oncol 20: 1489–1498. 19474112 10.1093/annonc/mdp033 PMC2731016

[28] Coleman RE, Banks LM, Girgis SI, Vrdoljak E, Fox J, et al (2010) Reversal of skeletal effects of endocrine treatments in the Intergroup Exemestane Study. Breast Cancer Res Treat 124: 153–161. 20730486 10.1007/s10549-010-1121-7

[29] Cuzick J, Sestak I, Baum M, Buzdar A, Howell A, et al (2010) Effect of anastrozole and tamoxifen as adjuvant treatment for early-stage breast cancer: 10-year analysis of the ATAC trial. Lancet Oncol 11: 1135–1141. 21087898 10.1016/S1470-2045(10)70257-6

[30] Coleman RE, Banks LM, Girgis SI, Kilburn LS, Vrdoljak E, et al (2007) Skeletal effects of exemestane on bone-mineral density, bone biomarkers, and fracture incidence in postmenopausal women with early breast cancer participating in the Intergroup Exemestane Study (IES): a randomised controlled study. Lancet Oncol 8: 119–127. 17267326 10.1016/S1470-2045(07)70003-7

[31] Thurlimann B, Keshaviah A, Coates AS, Mouridsen H, Mauriac L, et al (2005) A comparison of letrozole and tamoxifen in postmenopausal women with early breast cancer. N Engl J Med 353: 2747–2757. 16382061 10.1056/NEJMoa052258

[32] Hadji P, Gnant M, Body JJ, Bundred NJ, Brufsky A, et al (2012) Cancer treatment-induced bone loss in premenopausal women: a need for therapeutic intervention? Cancer Treat Rev 38: 798–806. 22429722 10.1016/j.ctrv.2012.02.008

[33] Fogelman I, Blake GM, Blamey R, Palmer M, Sauerbrei W, et al (2003) Bone mineral density in premenopausal women treated for node-positive early breast cancer with 2 years of goserelin or 6 months of cyclophosphamide, methotrexate and 5-fluorouracil (CMF). Osteoporos Int 14: 1001–1006. 14530912 10.1007/s00198-003-1508-y

[34] Gnant MF, Mlineritsch B, Luschin-Ebengreuth G, Grampp S, Kaessmann H, et al (2007) Zoledronic acid prevents cancer treatment-induced bone loss in premenopausal women receiving adjuvant endocrine therapy for hormone-responsive breast cancer: a report from the Austrian Breast and Colorectal Cancer Study Group. J Clin Oncol 25: 820–828. 17159195 10.1200/JCO.2005.02.7102

[35] Gallagher JC (2007) Effect of early menopause on bone mineral density and fractures. Menopause 14: 567–571. 17476146 10.1097/gme.0b013e31804c793d

[36] Shuster LT, Rhodes DJ, Gostout BS, Grossardt BR, Rocca WA (2010) Premature menopause or early menopause: long-term health consequences. Maturitas 65: 161–166. 19733988 10.1016/j.maturitas.2009.08.003 PMC2815011

[37] Ismail AA, O'Neill TW, Cockerill W, Finn JD, Cannata JB, et al (2000) Validity of self-report of fractures: results from a prospective study in men and women across Europe. EPOS Study Group. European Prospective Osteoporosis Study Group. Osteoporos Int 11: 248–254. 10824241 10.1007/s001980050288

[38] Nevitt MC, Cummings SR, Browner WS, Seeley DG, Cauley JA, et al (1992) The accuracy of self-report of fractures in elderly women: evidence from a prospective study. Am J Epidemiol 135: 490–499. 1570815 10.1093/oxfordjournals.aje.a116315

[39] Honkanen K, Honkanen R, Heikkinen L, Kroger H, Saarikoski S (1999) Validity of self-reports of fractures in perimenopausal women. Am J Epidemiol 150: 511–516. 10472951 10.1093/oxfordjournals.aje.a010040

[40] Wen CP, Levy DT, Cheng TY, Hsu CC, Tsai SP (2005) Smoking behaviour in Taiwan, 2001. Tob Control 14 Suppl 1i51–55. 15923450 10.1136/tc.2004.008011 PMC1766183

[41] Hershman DL, McMahon DJ, Crew KD, Shao T, Cremers S, et al (2010) Prevention of bone loss by zoledronic acid in premenopausal women undergoing adjuvant chemotherapy persist up to one year following discontinuing treatment. J Clin Endocrinol Metab 95: 559–566. 20022990 10.1210/jc.2009-1366 PMC2840866

[42] Kim JE, Ahn JH, Jung KH, Kim SB, Kim HJ, et al (2011) Zoledronic acid prevents bone loss in premenopausal women with early breast cancer undergoing adjuvant chemotherapy: a phase III trial of the Korean Cancer Study Group (KCSG-BR06–01). Breast Cancer Res Treat 125: 99–106. 20922564 10.1007/s10549-010-1201-8

