# Correction: Fracture in Asian Women with Breast Cancer Occurs at Younger Age

**DOI:** 10.1371/annotation/da898acf-fd59-480d-bea1-c91d13f43a5c

**Published:** 2013-09-23

**Authors:** Chun-Hao Tsai, Chih-Hsin Muo, Huey-En Tzeng, Chih-Hsin Tang, Horng-Chang Hsu, Fung-Chang Sung

The name of the first author is incorrect. The correct name is: Chun-Hao Tsai. The correct citation is: Tsai C-H, Muo C-H, Tzeng H-E, Tang C-H, Hsu H-C, et al. (2013) Fracture in Asian Women with Breast Cancer Occurs at Younger Age. PLoS ONE 8(9): e75109. doi:10.1371/journal.pone.0075109. 

